# Optimizing fitness for duty and post-combat clinical services for military personnel and combat veterans with ADHD—a systematic review of the current literature

**DOI:** 10.3402/ejpt.v5.23894

**Published:** 2014-08-14

**Authors:** Iliyan Ivanov, Rachel Yehuda

**Affiliations:** 1 Department of Psychiatry, Icahn School of Medicine at Mount Sinai, New York, NY, USA; 2 Department of Psychiatry, James J. Peters VA Medical Center, Bronx, NY, USA

**Keywords:** ADHD, military, PTSD

## Abstract

**Background:**

Attention deficit hyper activity disorder (ADHD) is a developmental disorder, most often diagnosed in childhood, and characterized by hyperactivity and inattention that negatively impacts one's ability to function and fulfill social and personal obligations. Individuals with past history of ADHD may enlist in the military under certain conditions, however the full impact of military training and deployment of later in life ADHD symptoms is unclear. It is of particular interest how military experience may affect ADHD in remission and if such individuals might be at elevated risk for relapse of ADHD symptoms.

**Method:**

We performed a systematic review f the available literature including the Department of Defense (DOD) guidelines for both eligibility to enlist and fitness for deployment based on reported history and current symptomatology of ADHD.

**Results:**

The after care for veterans with ADHD relapse is inconsistent and presents with number of challenges. We evaluate the DOD policies regarding the implications of ADHD for fitness for military service and post-combat mental health.

**Conclusion:**

The full extend of the interaction between pre-existing ADHD and post-combat PTSD are not fully understood. The development of comprehensive and clear algorithms for diagnosing and treating ADHD in the military before and after deployment will have a strong positive impact on the quality of care delivered to soldiers and veterans.

Attention deficit hyper activity disorder (ADHD) is a developmental disorder, most often diagnosed in childhood, and characterized by hyperactivity and inattention that negatively impacts one's ability to function and fulfill social and personal obligations. The natural course of the disorder may follow two different trajectories. In some cases, the symptoms spontaneously remit to the level of not affecting function by early adulthood. In other cases, the symptoms may persist from childhood to adolescence to adulthood and continue to compromise function. ADHD can be successfully treated with both pharmacological and behavioral interventions; however, treatment gains are generally short-lived. Moreover, those who experience continuous ADHD symptoms need to continue treatments with stimulant and non-stimulant medications. There is still a debate in the literature regarding the impact of past, presumably resolved ADHD, as well as current but successfully managed ADHD on functioning and mental health characteristics in adulthood. This debate has significance for military personnel during training and deployment as well as combat veterans seeking treatment following deployment. The Department of Defense (DOD) has clear guidelines for both eligibility to enlist and fitness for deployment based on reported history and current symptomatology of ADHD. While those with a history of ADHD may actually fair quite well during military training or when in non-combat roles, combat exposure may increase vulnerability for relapse of ADHD and other psychiatric conditions. This paper evaluates the DOD policies regarding the implications of ADHD for fitness for military service and post-combat mental health via a systematic review of the available literature since 2000. We also use illustrative case studies to highlight special clinical considerations associated with past or current ADHD in the context of other deployment-related conditions such as posttraumatic stress disorder (PTSD), and mild traumatic brain injury (mTBI) among others that may further complicate readjustment. As several neurobiological mechanisms have been related to ADHD symptoms, we will first discuss ADHD developmental trajectories and neurobiology.

## Developmental trajectories of ADHD and their effects on adult functioning

ADHD is among the most prevalent developmental disorders in childhood (e.g., 3–5%, Rowland et al., 2013; Visser et al., 2014). Some reports suggest that as high as 8% of youth 4–17 years of age in the United States are diagnosed with ADHD, and that approximately 4.5% have been diagnosed and treated for ADHD with either methylphenidate or amphetamine (Mayes et al., 2008). Of these youths, as many as 50% will continue to have some level of impairment in adulthood (van Lieshout, Luman, Buitelaar, Rommelse, & Oosterlaan, 2013) and 35% may continue to have a full persistence of symptoms. Only 6% will show remittance of problems with continuing treatment (Biederman et al., 2011). Long-term follow-up studies have shown that individuals with childhood ADHD have significantly worse educational, socioeconomic, and occupational outcomes as adults (Klein et al., 2013). Longitudinal studies in children have shown that both childhood (Kessler et al., 2006) and lifetime diagnosis of ADHD as well as problems with hyperactivity, antisocial behavior, and difficult temperament during childhood (Koenen et al., 2007) are associated with increased likelihood of exposure to trauma. Moreover, youths with ADHD are more prone to sustain severe injuries (Brehaut et al., 2003; Lam, 2002; Silva, Colvin, Hagemann, Stanley, & Bower, 2014) and that adolescents and adults with ADHD are more likely to have traffic violations and motor vehicle accidents relative to drivers without ADHD (Barkley, 2004).

The available evidence suggests that childhood ADHD follows two distinct clinical trajectories. About 50% go on to experience symptoms that will negatively affect adult functions (e.g., persisters). The other 50% will show remission of clinically meaningful symptoms of the disorder. It should also be noted that for persisters the presentation of symptoms changes with age so that overt hyperactivity diminishes whereas inattention presents itself, such as difficulties with organization, time management, and procrastination. Further, ADHD symptoms that persist in adulthood are possibly related to some persistent neurobiological perturbations. Such biological perturbations have been studied in respect to brain morphology and functions as well as brain neurochemistry since agents that affect dopamine and norepinephrine have been used successfully to remediate ADHD symptoms, as described below.

## Neurobiology of ADHD

The positive clinical effects of stimulants such as methylphenidate and amphetamines have drawn attention to the role of monoamines (e.g., noradrenaline and dopamine) in the pathophysiology of ADHD. More specifically, noradrenaline affects postsynaptic alpha (2A)-receptors in the prefrontal cortex (PFC) resulting in strengthening the connectivity among neurons while dopamine affects D (1) receptors, resulting in reduction of the “noise” from environmental input (Arnsten, 2009). PFC is critical for the regulation of behavioral responses, particularly for inhibiting inappropriate emotions, impulses and habits, and planning complex behaviors that require multiple interconnected responses. Children with ADHD show prominent abnormalities in the inferior PFC and its connections to striatal, limbic, cerebellar, and parietal regions (Arnsten & Rubia, 2012). In turn, drugs that facilitate the interconnections between the PFC and these other brain regions are expected to ameliorate ADHD symptoms.

Numerous studies have linked abnormalities in brain morphology, functions, and connectivity to hyperactivity and inattention observed in ADHD. For instance, morphological magnetic resonance studies have shown delayed cortical maturation (i.e., cortical thinning) in children with ADHD versus non-affected controls (Shaw et al., 2007; Sowell et al., 2003) so that brain changes that occur in healthy youth by ages of 10–12 are delayed by about 3 years for children with ADHD. One longitudinal neuroimaging study (Castellanos et al., 2002) showed reduced volumes in several brain regions (e.g., putamen and cerebellum) in children with ADHD compared to age-matched controls. Of interest is that some of these differences in cortical thickness and regional volumes disappear by late adolescence, which may account for the remission of ADHD symptoms in some individuals; conversely, symptom persistence may be associated with persistent morphological differences between ADHD and non-ADHD individuals in adulthood (Kasparek, Theiner, & Filova, 2013; Proal et al., 2011). Moreover, emerging evidence suggest that some of these morphological differences are diminished in individuals who receive medication treatments at the time of the scanning (Ivanov et al., 2010; Ivanov, Murrough, Bansal, Hao, & Peterson, 2014) providing further support for the idea that morphology and clinical phenotype are related. Changes in neuronal plasticity, which is modulated by the dopaminergic system, may be in the background of these differences in brain morphology and connectivity and methylphenidate treatment can lead to diminished morphological differences by its positive effect on neuroplasticity and connectivity (Kasparek et al., 2013; Peterson et al., 2009).

Understanding the developmental trajectories of ADHD and related neurobiology is important with respect to understanding the possible relationships of ADHD with other disorders that are highly prevalent in the military namely PTSD and TBI. As noted above, a considerable number of individuals experience reduction and remission of ADHD symptoms by late adolescence/early adulthood. It will be important, however, to examine if conditions associated with military service may affect the trajectory ADHD and via what mechanisms. It stands to reason to ask if remitted ADHD symptoms will relapse if an individual develops PTSD or is exposed to traumatic brain injury since these disorders often present with impaired attention.

One developmental model of ADHD suggests that ADHD symptoms are related to non-cortical (i.e., limbic system) dysfunction that manifests early in life and persists throughout a lifetime (Halperin & Schulz, 2006). Such dysfunctions could be potentially corrected by the influence of cortical structures (e.g., PFC) that function to regulate behavior as the neural and functional development of PFC during adolescence also parallels recovery from ADHD. Therefore, it stands to reason to conclude that for ADHD remitters the maturing prefrontal cortex modulates and compensates for the dysfunctions of limbic structures. What is important for individuals with ADHD in the military is how trauma (both physical and psychological) and subsequent PTSD might affect the compensatory interplay between PFC and non-cortical structures and if trauma can lead to possible ADHD relapse.

**Fig. 1:**
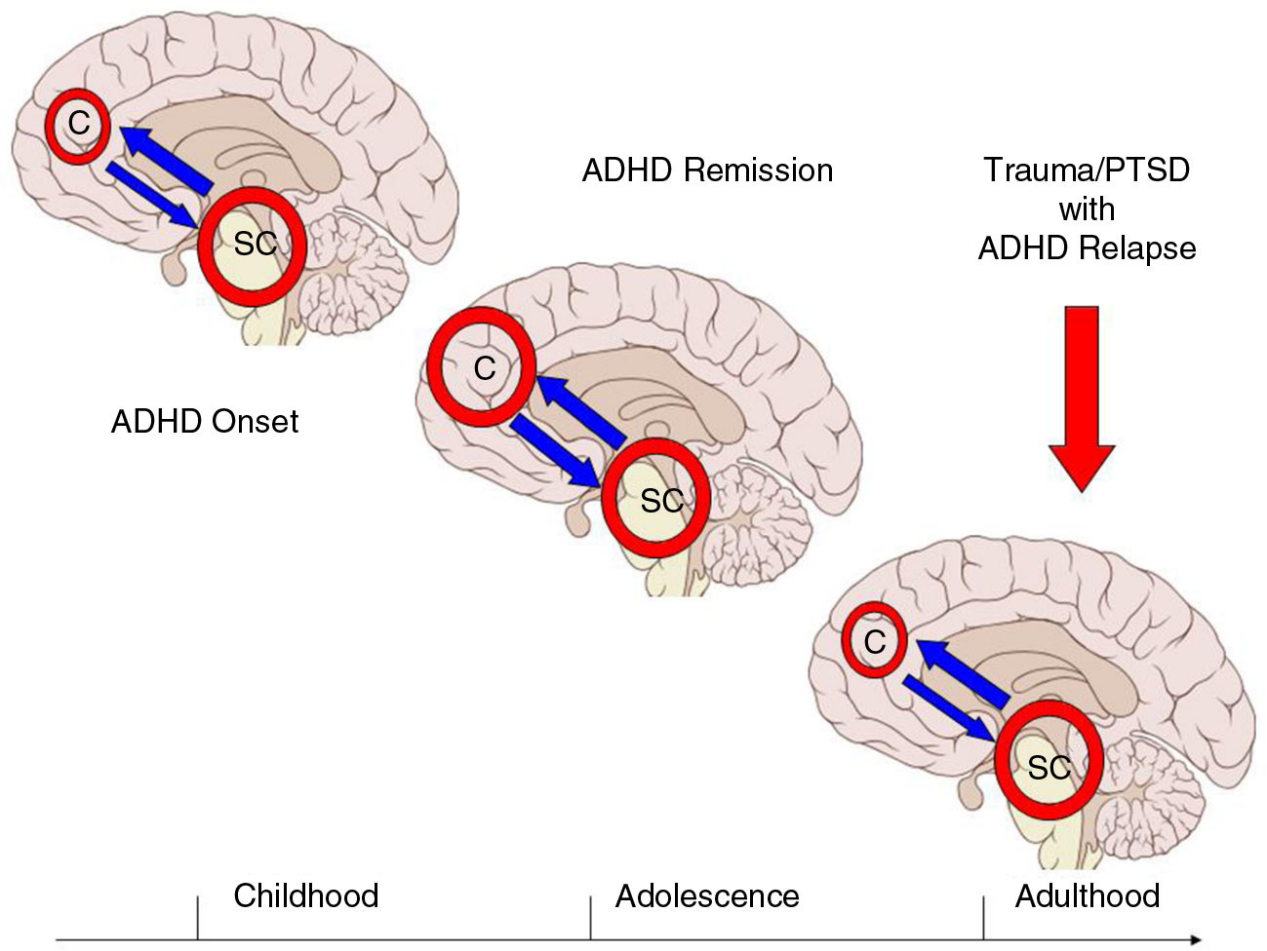
Model for the effects of trauma on cortical–subcortical functions and possible relapse of ADHD in adults with PTSD. Abbreviations: C=cortical; SC=subcortical. The figure shows hyperactive SC brain regions in childhood (i.e., ADHD onset) with subsequent compensation of C brain regions during adolescence that may be associated with remission of ADHD symptoms. In the event of military-/combat-related trauma and possible PTSD in adulthood, the compensatory effects of C brain regions might be reversed/compromised and ADHD symptoms may relapse.

The current neurocircuitry model of PTSD suggests that trauma causes dysfunction between hyperactive limbic regions (e.g., amygdala and dorsal part of the anterior cingulate) and hypoactive medial prefrontal regions (Hughes & Shin, 2011; Shvil, Rusch, Sullivan, & Neria, 2013). This type of circuitry disruption might be similar to the developmental functional mismatch between cortical and non-cortical regions (presenting as childhood ADHD). In a sense, trauma and PTSD can compromise the compensatory effects of the PFC and thus produce a relapse of ADHD symptoms as illustrated in Fig. 1. Alternatively, PTSD has been associated with dysfunctions of the dorsolateral prefrontal cortex so that the latter is sensitized to negative and threatening stimuli. (Fani et al., 2012) Such attention bias can produce states of hyper alertness and interfere with one's ability to attend equally to various environmental cues, which in turn will manifest as inattention.

Differentiating between relapse of ADHD versus inattention due to PTSD-associated hypervigilance and other non-ADHD factors (e.g., traumatic brain injury) will have implications for treatment of overlapping and unique features of ADHD versus comorbidities. As effective as stimulants can be for the reduction of ADHD symptoms (Arnsten & Pliszka, 2011; Gamo & Arnsten, 2011; Spencer, Klein, & Berridge, 2012), these agents can have negative effects on anxiety associated with PTSD. Moreover, it is unclear how these relapse symptoms will respond to available treatments in the presence of comorbid PTSD or TBI. Thus, it is important to consider some questions with respect to how ADHD might affect persons in the military. First, would such individuals be able to perform their duties to the required standards and second, what would be the effects of a military environment on the course of ADHD during and after active service?

## ADHD in military personnel

There is little evidence reporting on the prevalence of ADHD in particular occupations with the exception of one study that compared rates of ADHD in deploying soldiers and non-military counterparts (Hanson et al., 2012) showing no significant differences between these groups. In general, military training is highly structured and might provide a positive environment for ADHD as it has been shown that interventions that promote organizational skills and enhanced environmental structure are highly efficacious in adult ADHD (Solanto et al., 2010). This hypothesis is supported by a report from Rice, Butler, & Marra (2013) that evaluated active symptoms of ADHD and oppositional defiant disorder in soldiers during training and showed that these have minimal to no impact of the soldiers’ performance. One retrospective cohort study examined the rates of retention, promotion, and mental-health-related outcomes in 539 recruits with a history of ADHD and 1,617 control subjects for a period of 5 years (e.g., 1995–2000, Krauss, Russell, Powers, & Li, 2006). The results suggest that individuals with a history of ADHD can function efficiently in military service and be compatible to soldiers without such history (Krauss et al., 2006). It is necessary, however, to distinguish between different environments that may have notably different effects on one's functioning in relation to ADHD. As training periods may be characterized as relatively stable in respect to routine and schedules, these environmental factors are significantly different during deployment as the latter might be characterized as more unpredictable and demanding swift adaptations to ever-changing circumstances. Not surprisingly, soldiers who were previously diagnosed with ADHD showed 57% in-theater relapse rate of ADHD symptoms, which was higher than the relapse rates for previously diagnosed PTSD (55%), anxiety disorders (44%), mood disorders (38%), and adjustment disorders (32%) (Larson et al., 2011). Therefore, as soldiers with ADHD seem to be able to adequately adjust and participate in training exercises and activities (possibly due to the fact that these are highly structured), transitions from training to deployment and combat may be more challenging, may be due to greater unpredictability and the chaotic nature of combat events.

## ADHD and fitness for military service

DOD polices for ADHD as a criterion to enlist in voluntary military service has been influenced to some extent by the available data. However, many questions related to the influence of ADHD on performance during military service, deployment and post-deployment remain inadequately addressed. The above mentioned report by Krauss et al. (2006) documented no difference in the rates of retention, promotion, and mental-health-related outcomes between soldiers with and without ADHD; which in turn lead to changes in the DOD medical accession standards to allow applicants who reveal a history of ADHD, but did not require medication to finish high school or to hold a job for at least 1 year, the opportunity to enter active duty without a special waiver. However, more research in this area is needed.

It is worth noting that there are two sets of DOD criteria. The first are criteria to determine a person's fitness to enlist voluntarily for military service. The second are criteria to determine a soldier's fitness for deployment. The first set of criteria stipulate that if an individual has been able to maintain a steady functioning after the age of 14 that did not require special academic or vocational accommodations and did not need medication treatment with a single dose of medication longer than 24 months, then the person should be allowed to enlist. These criteria are shown on Table 1. The underlying premise appears to be guided by the principle of establishing an equal baseline for all enlisting soldiers so that they should be able to meet all the physical and cognitive requirements without the need for any special arrangements. These recommendations are also trying to strike a balance between not discouraging individuals to disclose history of ADHD by, on the one hand, not defining the condition as an automatic disqualifier and limiting the requirements for a waiver to join, but on the other hand, establishing guidelines that will prevent individuals with moderate to severe symptoms to enlist. The very important question of how to monitor the functioning of soldiers with ADHD at both base training and in-theater needs to be answered on the basis of empirical evidence currently lacking. The current DOD criteria for enlisting aim not to discriminate against individuals with a history of ADHD as long as they can demonstrate stable functioning with minimal medical care. As care is available during base training, individuals who may experience any symptom relapse/exacerbations should be evaluated and offered medication treatments if indicated. The assessment of their functional capacity should follow the established clinical practices of monitoring of adults with ADHD in civilian life. Special evaluations may need to be conducted to determine soldier's fitness for deployment similar to cases of any other medical condition.

**Table 1:** Criteria for enlisting in the military (as per Memorandum, Policy Guidance for Deployment-Limiting Psychiatric Conditions and Medications. Washington, DC, US Department of Defense, Assistant Secretary of Defense for Health Affairs, Nov 7, 2006) Applicants with history of ADHD may not enlist unless the following criteria are met:

Item #	Criteria
1	The applicant has not required an Individualized Education Program or work accommodations since the age of 14
2	There is no history of comorbid mental disorders
3	The applicant has never taken more than a single daily dosage of medication or has not been prescribed medication for this condition for more than 24 cumulative months after the age of 14
4	During periods off of medication after the age of 14, the applicant has been able to maintain at least a 2.0 grade point average without accommodations
5	Documentation from the applicant's prescribing provider that continued medication is not required for acceptable occupational or work performance
6	Applicant is required to enter service

A memorandum by the Assistant Secretary of Defense (2006) establishes the criteria for deployment. DOD memorandum declares conditions such as schizophrenia and bipolar disorder as automatic disqualifiers for deployment—as for other conditions, including ADHD, the criteria require that the soldier will have to be functionally stable for at least 3 months. Additional considerations in determining fitness for deployment include the need for medication treatments. Stimulants are the first line of pharmacological treatment for ADHD; they are also controlled substances and as such they are deemed as potentially problematic during deployment due to issues of storage, procurement, and abuse potential.

Despite these, further and more detailed investigations are needed to determine what might be the full extent of the relationship between ADHD symptoms and the highly stressful environments of the military service. It stands to reason to suggest that it is appropriate to establish more stringent criteria for deployment over enlistment while considering the fundamental differences between training and deployment in relation to environmental stress level and unpredictability. To summarize, it appears appropriate to have two different sets of guidelines that can address adequately the differences in environmental factors that can impact the course of neuropsychiatric disorders like ADHD. It seems appropriate to have somewhat stricter criteria for fitness for deployment; however, it should be noted that further narrowing of such criteria may unintentionally lead to under-reporting. Conversely, criteria that is too-loose lead to missed opportunities for appropriate diagnosing and treatment. In this next section, we will review the available literature on ADHD among military populations.

## ADHD in military personnel during post-deployment

Studies that examined the association between pre- and post-deployment mental disorders (Taubman, 2009) found that the recurrence of previously diagnosed disorders was common for service personnel who had received a psychiatric diagnosis before deploying; these individuals were also more likely than their counterparts with no pre-deployment diagnosis to receive a psychiatric diagnosis post-deployment. The high in-theater relapse rate of ADHD symptoms (see above Larson et al., 2011) poses the question as to what types of comorbid mental disorders may affect post-deployment readjustment for veterans. One report by Adler et al. (2004) found that veterans with PTSD reported higher levels of childhood ADHD relative to veterans with other anxiety disorder (e.g., panic disorder) and further speculate that ADHD may increase vulnerability for developing PTSD after trauma exposure. Similarly, Lee et al. (2012) report that current symptoms of ADHD represent an independent risk factor of symptoms of PTSD in Korean conscripts. More recently, a study in 222 male and female military show that 54.5% of the participants met the Diagnostic and Statistical Manual of Mental Disorders, Fourth Edition criteria for current PTSD. Of those, 11.5% also met the criteria for current adult ADHD; moreover, the severity of ADHD was a significant predictor of current PTSD severity (Harrington et al., 2012). Although not extensive, this evidence suggests a possible relationship between these two disorders such that a developmental condition such as ADHD may be seen as a predisposing factor for the later development of PTSD.

A few reports have examined the PTSD and ADHD comorbidity in both children and adults in terms of overlapping and unique clinical factors. Ford et al. (2000) found that children with ADHD did not differ in their hyperarousal symptom levels from their PTSD counterparts after accounting for overlapping symptoms between ADHD and PTSD (i.e., concentration problems). Adult studies suggests that the proportion of shared variance underlying PTSD-ADHD comorbidity is related to problems with modulating arousal levels that are common to both disorders (Harrington et al., 2012) and also show that comorbidity of PTSD and ADHD in adults is associated with greater clinical severity in terms of psychosocial functioning (Antshel et al., 2013). If it is true that persons with ADHD might be more likely to develop PTSD, then veterans with ADHD might be particularly vulnerable to the challenges of post-deployment adjustment.

The purported relationship between preexisting ADHD and late-in-life PTSD poses some serious challenges for the post-deployment rehabilitation of veterans. First, clinicians should consider the possibility that any particular sets of problems may not be exclusively related to a singular disorder (e.g., ADHD) but could be shared by other conditions. It has been shown that behaviors like distractibility, poor attention to tasks, frustration with difficult tasks and alike are present in children with trauma or abuse (Samuelson, Krueger, Burnett, & Wilson, 2010; Weber & Raynolds, 2004). Further, symptoms of hopelessness and uncertain future (as often reported in PTSD) could present as procrastination whereas hyper vigilance, feeling constantly on guard and experiencing frequent flashbacks could present as switching of activities and inability to complete tasks and assignments. Therefore, it is particularly important that clinicians will be familiar with the natural course and overlapping and unique features of ADHD and comorbid conditions (e.g., PTSD, TBI) that may mimic these types of symptoms. Next, we present composite clinical vignettes that aim to illustrate the above considerations.

M is a 26-year-old female who served in the navy and regards her years of service as “very rewarding and successful”—she describes the regimented work shifts as long but easy to navigate, and reports that she has excelled in her job and was highly regarded for her ability to complete her work on time. Psychiatric history is significant for reports of academic problems during middle and high school and self-reports of being “disorganized” and “not knowing how to complete my work” since “I used to do million things at once and finish none.” M reports that these problems caused her to fail classes and to miss deadlines including those for submitting her college application; however, by her account these problems “disappeared” during her military service—“even when I forget there was always someone to remind me what to do.” Toward the end of the tour, she experienced a significant personal loss and trauma as her best friend at work was killed in a gruesome accident and she had to “clean up and dispose of body parts.”

Shortly after M left the service to pursue different goals: “I wanted to take care of my son, to go to college and then find a well-paid job.” Upon discharge from the service she had a great deal of confidence in her abilities to achieve these goals. About a year later, she presented seeking help at the outpatient mental health clinic saying: “I am back to square zero.” She goes on to report that with time she found it extremely difficult to balance between maintaining employment, taking night classes, and attending to her son's care and upbringing. She said: “This is like high school all over again—I have so many ideas in my head but I don't know where to start and what to do first.” She also reports that when she needs to stay focused for extended time periods during class she finds herself thinking about her deceased friend and at times had “visions” of the accident. These symptoms became so debilitating that she cut down her work hours, was unable to pay her bills and moved back to live with her mother; she also failed most classes in the last semester of college.

This example describes preexisting attentional problems (presumably due to childhood ADHD) that may have been positively influenced by military environment to only re-occur with environmental change and may have been further influenced by military-related trauma. Establishing the presence of attention problems before the military service supports the diagnosis of ADHD independent of possible PTSD. In contrast, the onset of attention problems following focal trauma in the absence of symptoms in childhood/adolescence is suggestive of PTSD with attention problems independent of ADHD (see case below).

N is a 29-year-old male who served in the Army and was deployed overseas on combat mission. He has a sense of fulfilled duty but also feels very conflicted about his combat experience as he lost close friends and also “I saw things that I did not sign up for.” After military discharge he returned to his home town to live with family and started running his own private business—however, he gradually started losing interest in managing the business “the way I should,” spends less time at work, delegates most of the work to his associates and has no patience to go over the details of how to sustain and make the business grow. He finds it difficult to read long passages and to comprehend balancing his accounts. “I used to love books and was great at math in high school.” He often finds himself distracted from business activities either by thoughts about his deployment or “visions” when he sees people who he was fighting against. “We shoot at them and kill them but then they get up and stare at me.” He also describes similar types of dreams and on days after such nightmares he finds it particularly difficult to get out of bed and follow his daily routine.

## Detecting ADHD in treatment-seeking veterans

Many factors may account for symptoms of poor attention and distractibility in veterans who will seek treatment in the VA system. The VA has implemented a system of check lists and reminders that will prompt clinicians to look for symptoms of PTSD, TBI, suicidal behaviors, substance use and the alike. However, screening for ADHD is not high on this priority list and it is possible that inattention will be attributed to conditions other than ADHD. Undiagnosed and untreated ADHD may have a significant negative impact particularly for veterans who enroll for secondary education mainly because of the effects of ADHD on the ability to perform academic work. From a clinical standpoint proper identification of ADHD based on past and present history will inform a specific treatment plan and remediation strategies.

Given the current state of research into the symptom composition of ADHD and PTSD, a thorough clinical assessment remains the gold standard for proper differential diagnosis for veterans with trauma exposure and attention problems. As symptoms of ADHD may remit by late adolescence/early adulthood, “remission” does not always mean a full disappearance of these symptoms but rather changes in persons’ experience (Solanto et al., 2010). Adults with ADHD may not be hyperactive in the same fashion as young children but may experience an internal sense of restlessness, an inability to relax and need to move, which in turn could resemble and be misinterpreted as PTSD-related anxiety. In turn, high anxiety, preoccupation with intrusive thoughts, frequent flashbacks as well as effects of alcohol/drug use and the long-term effects of brain trauma may manifest as attention impairment. Differentiating between ADHD and PTSD may be crucial for treatment decisions since both stimulant and non-stimulant medications for ADHD may aggravate preexisting anxiety. In some cases, differentiating between ADHD and PTSD can be established over time via empirical treatment (i.e., restlessness due to ADHD may respond to ADHD medications when anxiety due to PTSD may respond to antidepressants or benzodiazepines). It is not unusual that a number of veterans seek continuation of mental health services that were initiated during active service, including prescribing of stimulant medications. However, prior treatment with stimulants does not confirm the diagnosis of ADHD because stimulants are not a treatment for the diagnostic entity of ADHD but rather affect and improve different components of impaired attention regardless of cause. In short, the establishment of adult ADHD diagnosis and disentanglement from other conditions related to exposure to traumatic stress during deployment could be rather challenging.

An important starting point will be the careful assessment of the nature and the chronology of symptom onset and presentation. By definition, ADHD is a developmental disorder with early childhood onset (Pliszka, 2007); however, individuals with predominantly inattentive symptoms may remain undiagnosed until later in life. A detailed interview should be able to elicit a pattern of attention problems with some level of functional impairment that precedes military service and possible military-related trauma. These patterns may include self-reports of underperformance in school, failing tests despite understanding the material (due to careless mistakes or easy distraction) and that homework assignments took too long to complete. Clinicians should ask about additional data such as available school records (e.g., report cards) with teachers’ impressions and recommendations; when possible an interview with a spouse/significant other could be conducted after obtaining patient's consent.

There are several scales that assess adult ADHD symptoms and their impact on functioning. These include Conners’ Adult ADHD Rating Scale (CAARS, Kooij et al., 2013) and the Adult ADHD Self-Report Scale (ASRS, Adler et al., 2006). These have shown consistent validity and test–retest reliability and can easily be administered in the office or at home. Similarly, scales that assess for the severity of PTSD symptoms, including PTSD Check List (PCL, Wisdom et al., 2013) and Clinician-Administered PTSD Scale (CAPS, Bauer et al., 2013) could be also used. Clinically, it might be useful to consider administering all scales with the assistance of the clinician; the benefit will be that clinicians can use appropriate prompts, thus reducing possible misinterpretation of the questions on the part of the patients. In addition, such instruments can be used pre and post treatment to assess intervention related improvement of symptoms. When possible, neuropsychological testing should be performed to rule out learning disabilities and to quantify specific attention deficits. However, the decision about neuropsychological testing should be outweighed with the understanding that such evaluation will take time and that it is not diagnostic for ADHD. All of these considerations need also to be clarified with the patient.

It is of paramount importance for clinicians to be able to carefully examine the associations between symptoms and particular settings and environmental influences. In short, while symptoms of ADHD are usually present from childhood (in some cases from pre-school years) and tend to manifest in multiple settings (i.e., work, school, social events), symptoms of PTSD are by definition related to either a focal trauma and chronic trauma exposure and are often associated and/or triggered by specific environmental factors. In Table 2, we present an algorithm for thorough clinical assessment of veterans for possible ADHD.

**Table 2:** Algorithm for the clinical evaluation for adult ADHD

Item #	Procedure
1	Establish the presence and severity of current ADHD symptoms
2	Establish age appropriate presentations—adults may report problems with time management, procrastination, planning, and completing tasks, and less overt hyperactivity
3	Establish the presence of these symptoms in multiple settings (e.g., work, school, family)
4	Establish the presence of negative impact of such symptoms on daily functioning (i.e., job performance is unsatisfactory, failing classes at school, bills unpaid, mismanagement of finances)
5	Inquire about prior treatments for similar problems (school/work accommodations, special mentorship, pharmacological treatments)
6	Establish the chronology of symptoms onset—childhood vs. adulthood
7	Establish triggers of adult symptom onset (i.e., symptoms started/ exacerbated after focal trauma)
8	Consider using self-report questionnaires (e.g., CAARS/ASRS for ADHD vs. CAPS/PCL for PTSD)
9	Establish possible comorbidities that may explain current symptoms (i.e., PTSD, mild TBI)
10	Inquire/establish past/current substance use problems
11	Consider consultations—neuropsychological testing to rule out learning disabilities, neurology for possible mild TBI

## Treatment considerations for ADHD in the context of PTSD comorbidity

Various treatments, both biological and psychosocial, have been developed to address the prominent symptoms of ADHD and PTSD. Cognitive-behavioral interventions should be considered and discussed for all cases of attention/cognitive deficits related to ADHD, PTSD or TBI in veterans. Clinicians should review learning styles and preferences, identify triggers and distractors and counsel patients on behavioral changes that could improve day-to-day functioning. These behaviors may include outlining preparatory steps before starting an assignment, using frequent breaks and time management charts, employing self-reminders by the use of electronic devices. It is also important to assess the baseline severity of functional impairment—in cases of ADHD the symptoms should be present in more than just one setting—and help patients to identify the most pressing problems and develop a personal “matrix” to assess possible treatment benefits (i.e., what are the things the patient wants to change most and in what time period).

Among the many factors influencing treatment recommendations, there are two important considerations: 1) clinicians assessment as to what is the impact of symptoms on person's functioning and 2) patient's attitude toward therapy versus medications. Existing evidence suggests that psychotherapy (i.e., “talk therapy”) was preferred over medications (e.g., sertraline) and that this preference was related to how the treatment was perceived to reduce PTSD symptoms (Chen, Keller, Zoellner, & Feeny, 2013). As psychotherapy treatment could be linked to elevation of anxiety or other forms of emotional distress, it is not associated with “side effects” in the same sense as psychopharmacological treatments. Therefore, there is a different cost–benefit analyses that psychiatrists have to account for when recommending pharmacological treatments.

FDA approved biological treatments for adult ADHD include stimulant (methylphenidate and amphetamine preparations) and non-stimulant (atomoxetine); alpha-2 agonists have been approved for children only although adult data are available. In addition, there is some evidence for clinical benefits in adult ADHD from bupropion, venlafaxine, modafinil and other drugs (De Sousa & Kalra, 2012). Similarly, pharmacological agents such as SSRI or beta-blockers seem moderately effective as a first-line treatment for PTSD but less so for military personnel (Tawa & Murphy, 2013). A substantial body of evidence supports benzodiazepine use in panic disorder and generalized anxiety that may frequently accompany PTSD (Ravindran & Stein, 2010).

The clinical decision to start pharmacotherapy should be based on the clinician's impression of how symptoms interfere with a patient's ability to maintain social functioning. In that respect, stimulants and benzodiazepines share some similarities; they both could be considered “rescue” medications due to their ability to quickly affect attention—in the case of stimulants—and anxiety in the case of benzodiazepines. Strong indications for initiating a stimulant trial would be the presence of attentional problems that seem to interfere with most aspects of person's life—social, professional, academic, and family. In such cases, the clinician has to balance between the purported benefits and the possible effect of the stimulant agents on symptoms of anxiety that may be related to comorbid PTSD. Common effects of stimulants as well as atomoxetine include activation of the peripheral autonomic system resulting in elevation of pulse and blood pressure, which might be experienced as psychological arousal. When such arousal might be beneficial in cases of ADHD (when there might be deficits in initiating action), these might have a different effect in persons with PTSD. The biology of PTSD is linked to persistent states of exaggerated sensitivity of various components of hypothalamic–pituitary axis (HPA) that patients may experience as states of high anxiety, hypervigilance, and recurrent panic attacks. These could be further exacerbated by pharmacological interventions aimed to benefit ADHD while on the contrary complicating comorbid PTSD. Therefore, it might be prudent to initiate stimulants at low doses and proceed with incremental dose changes; in general, possible side effects of stimulants are managed by discontinuation of the stimulant and in most cases these side effects are time limited. Similarly, as benzodiazepines could be very helpful with diminishing the intensity and the frequency of panic attacks, they may in turn exacerbate attention problems since psychomotor slowing is a common side effect of benzodiazepines.

The successful management of these types of problems is dependent on adequate psycho-education with respect to both expected benefits and potential limitations/side effects. The clinician must emphasize that these treatments, although potentially very effective, do not provide “cure” for these conditions. Second, the clinician and patient need to discuss short-term versus long-term treatments. For instance, both stimulants and benzodiazepines have considerable abuse potential so it is probably best not to prescribe them simultaneously for extended amount of time. The clinician also has to instruct and work with the patient to assure reliable monitoring of the drug by starting with limited supplies and encouraging patient to take full accountability of his medications. With certain exceptions, refills for “lost” or “missing” medications should be discouraged. In addition to detailed substance use history, clinicians should request random urine toxicology to identify co-occurring drug use. Third, patients should be counseled and educated on alternative agents. These may include non-stimulants (e.g., Atomoxetine, alpha (2A) receptor agonists) for ADHD and antidepressant SSRIs and gabaergic compounds (e.g., topiramate, gabapentin) for PTSD. These agents lack abuse potential and therefore do not require the strict monitoring that is the standard of care for stimulants and benzodiazepines. The above outlined topics could be illustrated with the following vignette.

K is a 28-year-old male requesting treatment for persistent attentional problems. He reports that in high school his grades are “just good enough to pass” and that he felt unmotivated and bored in class and “never did homework.” Although not formally diagnosed with ADHD, he remembers that teachers had encouraged his parents to seek psychological evaluation, which they never did. K enlisted in the military because “I knew I can never make it in college.” K was evaluated while in the service and was diagnosed with adult ADHD for which he received Adderall with positive effects. He reports that his work benefited as he felt more organized and focused. He also experienced focal combat-related trauma with subsequent flashbacks, nightmares, and panic attacks—the latter triggered specifically by airplane travel. After military discharge, he presented requesting continuation of treatment at the VA clinic stating that he needs Adderall in order to manage his academic work as he enrolled in college—the way the medicine helped with his military job suggested to him that he will be better able to do school work with the medicine. He was interested in therapy to resolve emotional issues related to his combat exposure and after several months of trauma-focused therapy, his flashbacks and nightmares have mostly resolved. As he continues to experience panic attacks specifically related to airplane travel (he traveled often for job interviews), he was prescribed a small amount of Clonazepam 1 mg tabs to take as needed for plane travel and these prescriptions were closely monitored. Taking into consideration the presence of anxiety symptoms, Adderall was changed to atomoxetine (Strattera) that was similarly beneficial and K was able to successfully complete his Bachelor's degree.

## Gaps in knowledge

Despite the progress, several areas related to the interplay between ADHD and PTSD in military service personnel remain poorly explored. Current recommendations for restricted duty are based on limited empirical data. Available evidence suggests that military personnel with preexisting and new-onset mental health conditions did not differ in the ability to perform their jobs. In addition, despite the high relapse rate of attentional and anxiety disorder during deployment, the majority of soldiers who seek mental health in-theater did not have known preexisting disorders, therefore factors other than prior mental health concerns clearly influence resilience during combat (Rona et al., 2009). On contrary, longitudinal research in ADHD has consistently shown long lasting negative consequences in non-military populations—these type of data are missing in respect to long-term outcomes post-military service and deployment. Considering findings that over 50% of the personnel with a pre-deployment psychiatric diagnosis had received the diagnosis in the 9 months before their encounter with mental health services, in-theater further study may be advisable to determine whether a minimum time should be determined between establishment of a diagnosis and eligibility for deployment and whether it should vary by diagnosis (i.e., ADHD vs. PTSD).

## Conclusion

In this paper, we reviewed DOD guidelines that allow for individuals with ADHD to enlist for military service. Preexisting ADHD may certainly affect soldiers’ participation in basic training and performance in theater. Under conditions of traumatic stress, individuals with ADHD may experience a relapse of ADHD symptoms as well as the development of new symptoms of inattention and other cognitive deficits that could be attributed to military-related PTSD. We suggest that proper understanding of the developmental aspects of ADHD and PTSD will inform the diagnostic procedures and will help clinicians to differentiate between these conditions. Further, clinicians’ knowledge about the neurobiological underpinnings of ADHD and PTSD should inform the choice of appropriate treatment strategies considering the use of both psychotherapy and psychopharmacological regimens alone or in combinations. The development of comprehensive and clear algorithms for diagnosing and treating ADHD in the military before and after deployment will have a strong positive impact on the quality of care delivered to soldiers and veterans.
